# Spontaneous Intracranial Hypotension Associated with Marfan Syndrome: A Case Report

**DOI:** 10.5811/cpcem.7223

**Published:** 2024-05-29

**Authors:** Faiza Tariq, Wesley Eilbert

**Affiliations:** University of Illinois, College of Medicine, Department of Emergency Medicine, Chicago, Illinois

**Keywords:** *spontaneous intracranial hypotension*, *Marfan syndrome*, *meningeal diverticulum*, *spinal cerebrospinal fluid leak*, *case report*

## Abstract

**Introduction:**

Spontaneous intracranial hypotension (SIH) is an uncommon and frequently misdiagnosed condition characterized by a lower-than-normal volume of cerebrospinal fluid (CSF) caused by leakage of CSF through the dural membrane. The primary manifestation of SIH is an orthostatic headache, which is frequently accompanied by nausea and vomiting. Patients with connective tissue disorders are at increased risk for spontaneous CSF leaks due to the structural weakness of their dural membranes.

**Case Report:**

An 18-year-old woman with no reported past medical history presented to the emergency department with 10 days of a bifrontal headache that was orthostatic in nature with associated nausea and vomiting. She was noted to have several marfanoid features on physical examination. Spontaneous intracranial hypotension was ultimately diagnosed and treated successfully with an epidural blood patch. Subsequent genetic testing revealed a diagnosis of Marfan syndrome.

**Conclusion:**

Spontaneous intracranial hypotension is an uncommon cause of headache. Individuals with connective tissue disorders such as Marfan syndrome are at increased risk for SIH. Knowledge of the relationship between these two conditions allows for a more rapid diagnosis of SIH.

Population Health Research Capsule What do we already know about this clinical entity?

*Spontaneous intracranial hypotension (SIH) is caused by leakage of cerebrospinal fluid through the dural membrane, which can be weakened by connective tissue disorders.*

What makes this presentation of disease reportable?

*A woman who presented with an orthostatic headache was ultimately diagnosed with SIH. Genetic testing later revealed a diagnosis of Marfan syndrome.*

What is the major learning point?

*Individuals with connective tissue disorders such as Marfan syndrome are at increased risk for spontaneous cerebrospinal fluid leaks resulting in SIH.*

How might this improve emergency medicine practice?

*Knowledge of the predilection of patients with connective tissue disorders for SIH may assist clinicians in the diagnosis of similar cases.*

## INTRODUCTION

First described in 1938, spontaneous intracranial hypotension (SIH) is a condition characterized by a lower-than-normal volume of cerebrospinal fluid (CSF) caused by leakage of CSF through the dural membrane.^1^ ^,^ ^2^ Patients with connective tissue disorders are at increased risk for spontaneous CSF leaks due to the structural weakness of their dural membranes.^3^ The primary manifestation of SIH is an orthostatic headache that may be accompanied by nausea, tinnitus, and photophobia.^4^ We report the case of a young woman with previously undiagnosed Marfan syndrome who presented with SIH.

## CASE REPORT

An 18-year-old woman presented to the emergency department (ED) complaining of a bifrontal headache present for 10 days. The headache was of insidious onset and was significantly worse in the upright position, better when supine. She reported nausea with the headache and one episode of vomiting two days earlier. On physical examination, she had several marfanoid features including a tall, slender build with disproportionately long arms, legs, and fingers. She was afebrile and had a normal neurologic examination.

Computed tomography of her brain showed no significant abnormalities and magnetic resonance imaging (MRI) of the brain with venogram was ordered because of concern for possible dural venous sinus thrombosis. The MRI showed dural thickening and increased dural enhancement suggestive of intracranial hypotension. The patient was admitted to the hospital, and MRI of the cervical, thoracic, and lumbar spine performed the next day showed no extradural fluid collections to suggest a CSF leak. She had modest improvement of her headache after an epidural blood patch and was discharged on hospital day three.

The patient returned to the ED five days later complaining of a return of the headache. A second MRI of the spine was performed that showed a meningeal diverticulum arising from the left first and second thoracic level with an adjacent pleural effusion concerning for a possible ruptured nerve root sleeve diverticulum (Image). She received a second epidural blood patch with complete, longstanding relief of the headache. Subsequent outpatient genetic testing revealed a pathogenic variant of the FBN1 gene consistent with a diagnosis of Marfan syndrome.

**Image.**
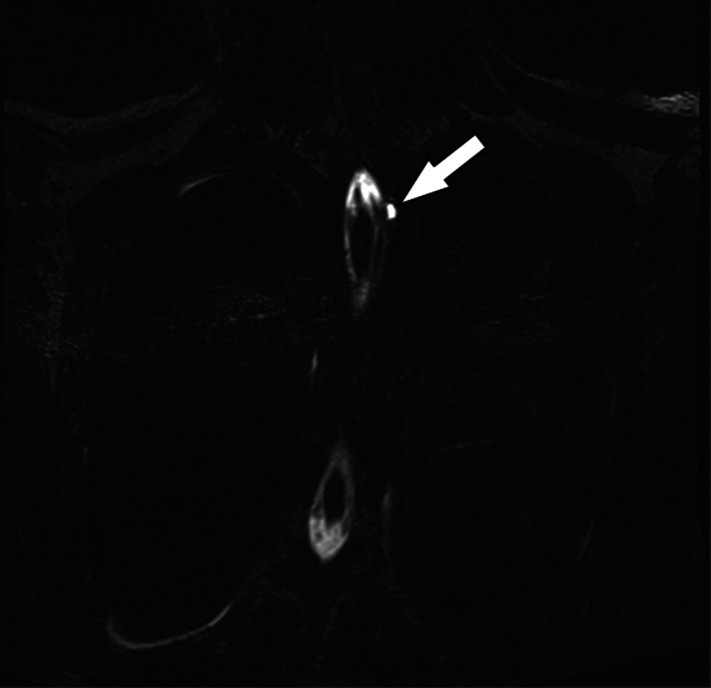
Magnetic resonance imaging (coronal view) showing a meningeal diverticulum at the first and second thoracic level (arrow).

## DISCUSSION

Spontaneous intracranial hypotension is distinguished from other causes of CSF leaks of known cause such as spinal surgery, craniospinal trauma, and, most commonly, lumbar puncture or spinal anesthesia. Spontaneous intracranial hypotension is an uncommon cause of headache in the ED, with an estimated annual incidence of 5/100,000 individuals—half the incidence of subarachnoid hemorrhage.^5^ Women are diagnosed with SIH more frequently than men, with a female-male ratio of approximately 2:1.^6^ Spontaneous intracranial hypotension is often initially misdiagnosed, with 94% of patients receiving an incorrect initial diagnosis in one study.^7^ The headache caused by SIH is believed to be the result of downward displacement of the brain due to loss of CSF buoyancy, resulting in traction on pain-sensitive fibers in the dura mater.^3^ ^,^ ^6^ The orthostatic headache caused by SIH is typically described as diffuse, occipital, or frontal.^4^ Other associated symptoms are often present (Table 1).^4^

**Table 1.** Symptoms associated with the headache of spontaneous intracranial hypotension.

Symptom	Estimated proportions
Nausea/vomiting	54%
Neck pain/stiffness	43%
Hearing disturbances	28%
Dizziness	27%
Tinnitus	20%
Vertigo	17%
Photophobia	11%

The location of the CSF leak with SIH is often not determined since spinal CSF leaks generally do not cause local symptoms.^6^ Extradural spinal CSF collections are found in approximately half of all patients with SIH.^2^ The majority of identified spinal CSF leaks occur at the thoracic or cervicothoracic spine level.^8^ Spinal CSF leaks occur via three main mechanisms: meningeal diverticula; ventral dural tears; and CSF-venous fistulas, with diverticula being most common.^9^ Patients with genetic connective tissue disorders such as Marfan syndrome, Ehlers-Danlos syndrome Type II, and autosomal dominant polycystic kidney disease are at higher risk for spontaneous CSF leaks due to the structural weakness of their dural membranes.^3^ ^,^ ^10^ One prospective study found that up to two-thirds of patients with spontaneous spinal CSF leaks had some evidence of a connective tissue disorder.^10^

Diagnostic criteria for SIH have been proposed (Table 2).^2^ ^,^ ^11^ Brain MRI with contrast is the most sensitive imaging study for diagnosing SIH.^4^ ^,^ ^9^ Smooth, generalized dural enhancement is the feature most commonly seen, present in 73% of patients with SIH.^2^ ^,^ ^4^ Many cases of SIH resolve spontaneously with no specific treatment required.^12^ Initial conservative treatments of bed rest, oral hydration, and caffeine are effective in approximately 25% of patients.^4^ Epidural blood patching is the most commonly performed intervention for spinal CSF leaks, with estimates of efficacy ranging from 36–90%.^9^

**Table 2.** Diagnostic criteria for spontaneous intracranial hypotension.

1. Any headache attributed to low CSF pressure or CSF leakage that meets criterion 3 below.
2. Either or both of the following: a. Low CSF pressure (<60 mm H_2_O CSF) b. Evidence of CSF leakage on imaging
3. Headache that developed in temporal relation to the low CSF pressure or CSF leakage, or that leads to its discovery.
4. Absence of a procedure or trauma known to be able to cause CSF leakage.
5. Headache not better accounted for by another diagnosis.

*CSF*, cerebrospinal fluid; *mm H_2_O*, millimeters of water.

## CONCLUSION

Spontaneous intracranial hypotension is an uncommon cause of headache and is frequently misdiagnosed. Individuals with Marfan syndrome and other connective tissue disorders are at increased risk for SIH due to the structural weakness of their dural membranes predisposing to spontaneous CSF leaks. Knowledge of the relationship between these associated conditions allows for more rapid diagnosis of SIH.
